# Nutrition and metabolic factors in cancer causation and prevention

**DOI:** 10.1186/2049-3002-2-S1-O6

**Published:** 2014-05-28

**Authors:** Elio Riboli

**Affiliations:** 1 School of Public Health, Imperial College London, UK

## 

EPIC represents a large and mature resource for epidemiological studies of cancer aetiology. Data can be integrated from studies on diet, physical activity, anthropometry and biomarker measurements to build a broad picture of the role of nutrition and metabolic factors in cancer causation. Since the initial baseline collection, cohort participants have been followed up to obtain information on changes in major dietary and lifestyle factors including a series of questions on whether the subjects had suffered from a number of common diseases and medical conditions. So far, over 60,000 newly incident cancer cases have been reported, including around 14,000 breast, 3,600 lung, 5,200 colorectal, 1,180 pancreatic, and 1,400 endometrial cancer cases, giving scope for well-powered studies on cancer causation. Some notable findings from EPIC research include the association between fibre consumption and a reduced risk in the development of colorectal cancer, red meat consumption and an increased risk of digestive tract cancers, positive associations with BMI and colon cancer in men, waist-hip ratio and the development of colon cancer in both men and women and a significant prediction between being overweight (expressed as BMI) and breast cancer risk in postmenopausal women not using HRT. It is clear that a number of chemical, physical and biological carcinogens have a major role in cancer causation and in some cases account for most of the variations in cancer incidence across the world. However, they cannot explain the global variations for a large number of cancers that represent a significant proportion of the world’s total cancer burden. Research conducted during past decades, and particularly those based on large prospective studies including EPIC, has found that diet, nutrition physical activity and anthropometry have major influences on population risk of cancer. From a scientific point of view, the unravelling of the mechanistic effects underlying the increased cancer risk associated with some components of current western diet and lifestyle is challenging. The effect may be due to fine modulation or modest dis-regulation of normal physiological processes rather than gross disruption, as may be the case for some strong exogenous carcinogens. From a public health point of view, these metabolic lifestyle factors are of great interest as they largely overlap with the major known risk factors of cardiovascular diseases and diabetes, therefore offering the opportunity of targeting the prevention of a number of non-communicable diseases with the same panel of public health interventions.

